# Fatal pulmonary thromboembolism associated with excessive nitrous oxide consumption over several weeks

**DOI:** 10.1007/s00414-026-03958-z

**Published:** 2026-08-12

**Authors:** Stefanie Plenzig, Friederike Pijahn, Alexander Müller, Stefan W. Toennes, Silvana Petzel-Witt

**Affiliations:** 1 Institute of Legal Medicine, Goethe University, University Hospital, Frankfurt, Germany; 2 Department of Legal Medicine, University Medical Center Hamburg Eppendorf, Hamburg, Germany

**Keywords:** Nitrous oxide, Hyperhomocysteinemia, Thromboembolic events, Vitamin B12 deficiency, Chronic drug misuse

## Abstract

A man in his early twenties had been hospitalized due to acute nitrous oxide intoxication, during which a vitamin B12 deficiency and anaemia were also diagnosed. Two weeks later he was found dead in his apartment. Twenty empty nitrous oxide cartridges were found next to the body, and according to his mother, he had been using nitrous oxide excessively for approximately two months. The autopsy revealed a fatal pulmonary embolism secondary to deep vein thrombosis, and toxicological analysis detected nitrous oxide in low concentrations in the blood. Regarding the pulmonary embolism, a coagulation disorder attributable to suspected hyperhomocysteinemia in the setting of vitamin B12 deficiency could be considered a contributory factor – this deficiency, in turn, may possibly be related to excessive nitrous oxide consumption over several weeks. Obesity and nicotine abuse were present as additional risk factors for thromboembolic events. Reapeted excessive nitrous oxide consumption can induce profound metabolic disturbances, including functional vitamin B12 deficiency and associated biochemical derangements. In cases of fatal thromboembolic events in young individuals, this possibility should be taken into account. Toxicological analysis may demonstrate recent nitrous oxide exposure and support the hypothesis, but it cannot establish the underlying biochemical pathology.

## Introduction

Nitrous oxide (N_2_O) is used clinically as an anaesthetic for children in dentistry [1]. Due to its low cost and hallucinogenic properties, it is increasingly being consumed as a party drug [2]. In the context of this purpose it is readily available through online shops and kiosks [3]. Several case reports now warn of potentially severe (neurological) sequelae and acute medical risks, such as thromboembolic events [2, 4–6]. To curb the misuse of nitrous oxide, the German Bundestag subsequently passed a bill in November 2025 to amend the New Psychoactive Substances Act (Neue-psychoaktive-Stoffe-Gesetz) for nitrous oxide [7].

A case is presented in which death occurred following a thromboembolic event associated with excessive nitrous oxide consumption over several weeks.

## Case report

A man in his early twenties had been hospitalized two weeks before his death due to acute nitrous oxide intoxication; he had consumed around four bottles of nitrous oxide a day over the previous eight days and, according to his mother, had become increasingly drowsy and had repeatedly fainted. On hospital admission, he presented with sinus tachycardia (120 bpm), paranoid behaviour and appeared generally lethargic. He had a vitamin B12 deficiency (120 pg/mL; normal range > 200 pg/mL) and a low haemoglobin level (10.5 g/dL; normal range 13.5–16.5 g/dL). The medical records did not indicate that any further parameters (e.g. homocysteine, mean corpuscular volume) had been measured. He had no other relevant known medical history and no known family history of relevant medical conditions. He was administered vitamin B12 parenterally and was discharged from hospital the following day.

**Fig. 1:**
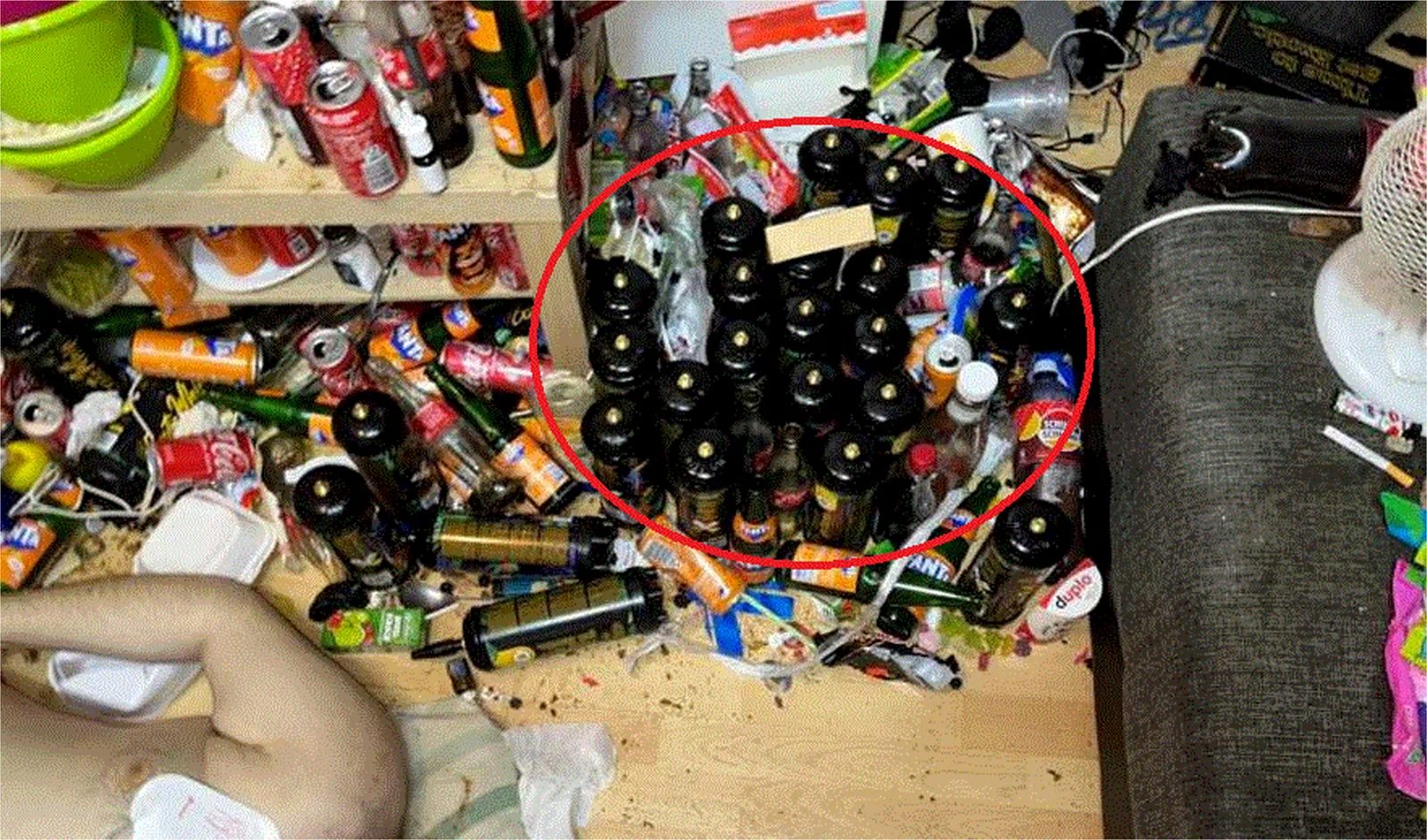
Nitrous oxide cylinders next to the corpse

Two weeks later he was found lifeless in his room by his mother in the morning. The mother alerted the emergency services and performed CPR under their guidance; this was initially continued by the paramedics - without success. The man had last been seen alive by his mother the previous evening. He had been using cannabis, smoking tobacco and drinking alcohol for several years. For approximately two months, he had been using nitrous oxide to a significant extent. Twenty bottles of ‘Exotic Whip’ nitrous oxide were found in the man’s room (Fig. 1), along with approximately 30 used rubber balloons in the immediate vicinity. His mother reported that he had trouble walking and often suffered from cold sweats. Furthermore, he had frequently trembled and suffered from bouts of weakness and panic attacks.

To determine the cause of death, a medicolegal autopsy was ordered, including toxicological analyses.

### Autopsy and histology

12 days after death, the corpse of the obese young man (height 1.79 m, weight 133 kg, BMI 41.5 kg/m^2^) was transferred to the Institute of Legal Medicine, where a full medicolegal autopsy was carried out. The main findings were deep vein thrombosis and fulminant pulmonary artery thromboembolism. Secondary findings included haemorrhagic pulmonary oedema and cerebral oedema. Histological examinations of all parenchymal organs were performed. Lungs displayed an intense haemorrhagic oedema and thrombi in the pulmonary arteries were seen.

### Toxicological analysis

Toxicological analysis detected caffeine in the gastric contents, heart blood, and bile. Urine testing revealed 35 ng/mL of THC-carboxylic acid, while femoral venous blood contained only trace amounts of THC and THC-carboxylic acid (< 1 ng/mL). Salicylic acid was present in heart blood, consistent with prior ingestion of acetylsalicylic acid. Nicotine and its metabolite were identified in heart blood and urine, indicating recent tobacco exposure. Ethanol was not detected in femoral venous blood and only a small concentration in urine (0.31 g/L). Screening for volatile substances in heart blood, lung tissue, and brain yielded negative results.

A targeted nitrous oxide analysis was performed with bronchial fluid and a separately obtained femoral venous blood sample using a validated HS-GC/MS (headspace - gas chromatography/mass spectrometry) method. After chromatographic separation from isobaric carbon dioxide on a 15 m PLOT column, nitrous oxide was detected in selected-ion mode (SIM) using n-pentane as the internal standard. Calibration covered a range of 0.01–10 mL/L with a lower limit of quantification (LLOQ) of 0.01 mL/L. In this case, nitrous oxide concentrations of 0.06 mL/L in bronchial puncture fluid and 0.04 mL/L in femoral venous blood clot were measured.

The presumed cause of death was acute right heart failure resulting from a fulminant pulmonary artery thromboembolism caused by deep vein thrombosis.

## Discussion

Nitrous oxide (N₂O) misuse has emerged as an increasingly recognized cause of neurological, haematological, and thrombotic complications. These complications are largely mediated through functional inactivation of vitamin B12 and subsequent metabolic derangements [8–10]. We report a fatal case in which excessive nitrous oxide consumption over several weeks may have contributed to the development of deep vein thrombosis and fatal pulmonary artery thromboembolism.

The deceased, a young adult male, was found in his residence, where approximately twenty nitrous oxide cylinders were present, including one located adjacent to the body. Toxicological analyses detected nitrous oxide concentrations of 0.06 ml/L in bronchial puncture fluid and 0.04 ml/L in femoral venous blood clot. Although these concentrations were low, they remained above atmospheric background levels [11] and within the analytical calibration range, indicating prior intake. Evidence suggests that nitrous oxide can still be detected in post-mortem samples for several weeks or even months [12, 13]. Given the high volatility of nitrous oxide, postmortem dissipation and losses during sampling can be expected; the measured values therefore likely underestimate the concentrations present at the time of death. However, the low concentrations are regarded as an indication of consumption that took place a few hours before death. According to the mother, the deceased had consumed nitrous oxide in large quantities every day for approximately two months.

The medical history revealed a hospital admission two weeks earlier due to acute nitrous oxide intoxication. Laboratory testing demonstrated a vitamin B12 concentration of 120 pg/mL (reference > 200 pg/mL) and a haemoglobin level of 10.5 g/dL, consistent with cobalamin deficiency and anaemia. Parenteral vitamin B12 was administered. No homocysteine or methylmalonic acid measurements were performed. Postmortem assessment of these metabolic markers was not feasible due to specimen limitations.

The pathophysiological consequences of chronic nitrous oxide exposure are well established [8, 10]. Nitrous oxide oxidizes the cobalt ion of cobalamin, irreversibly inhibiting methionine synthase and thereby disrupting on carbon metabolism [14]. This inhibition leads to impaired remethylation of homocysteine to methionine, resulting in hyperhomocysteinemia, endothelial dysfunction, and a prothrombotic state. This metabolic disturbance is a recognized risk factor for venous thromboembolism, acting through oxidative stress, reduced nitric oxide bioavailability, and a procoagulant endothelial phenotype [10]. Doukhi et al. [15] described two cases of cerebral venous thrombosis linked to nitrous oxide–induced hyperhomocysteinemia, underscoring the mechanistic plausibility of thrombotic complications in this context. Earlier systematic reviews, such as Garakani et al. [16], have highlighted the haematological and neurological sequelae of chronic nitrous oxide exposure, including megaloblastic anaemia and functional vitamin B12 deficiency [8].

In the present case, the documented vitamin B12 deficiency several weeks before death strongly suggests that metabolic disturbances persisted at the time of the fatal event. Although homocysteine levels were not available, the exposure history, clinical presentation, and known biochemical mechanisms support the presence of hyperhomocysteinemia. Additional risk factors, such as obesity and tobacco use, increase the thrombotic risk [17, 18]. In this specific case, a BMI of 41.5 kg/m^2^ and reported cigarette smoking may have further contributed to the development of deep vein thrombosis.

## Conclusion

Repeated excessive nitrous oxide abuse can induce profound metabolic disturbances, including functional vitamin B12 deficiency and associated biochemical derangements. In cases of fatal thromboembolic events in young individuals, this possibility should be taken into account. Toxicological analysis may demonstrate recent nitrous oxide exposure and support the hypothesis, but it cannot establish the underlying biochemical pathology.

## Data Availability

No datasets were generated or analysed during the current study.
